# Rehabilitation Workforce Interventions for Pressure Ulcer Prevention: A Scoping Review

**DOI:** 10.3390/nursrep16090339

**Published:** 2026-09-18

**Authors:** José Júlio Veríssimo, Júlio Belo Fernandes

**Affiliations:** 1 Unidade de Cuidados Continuados Integrados São Roque, Santa Casa da Misericórdia de Lisboa, 1769-001 Lisbon, Portugal; verissimo9@gmail.com; 2 Egas Moniz Center for Interdisciplinary Research (CiiEM), Egas Moniz School of Health & Science, 2829-511 Almada, Portugal; 3 Nurs* Lab, 2829-511 Almada, Portugal

**Keywords:** pressure ulcer prevention, rehabilitation interventions, rehabilitation workforce, self-care deficit theory, scoping review

## Abstract

**Background**: Pressure ulcers remain a prevalent and largely preventable adverse event across healthcare settings, particularly among people with impaired mobility and activity, and reduced self-care capacity. Although rehabilitation interventions address key risk factors for pressure ulcer development, their contributions are often implicit and fragmented in the literature. This review aims to systematically map rehabilitation interventions described in the literature for pressure ulcer prevention across healthcare settings. **Methods**: We conducted a scoping review following the Arksey and O’Malley framework and reported it according to PRISMA-ScR. Eight electronic databases were searched for studies published between 2015 and 2026 in English or Portuguese. Rehabilitation interventions aimed at preventing pressure ulcers were mapped and synthesised using Orem’s Self-Care Deficit Theory as an interpretive lens. **Results**: Eight records were included. Rehabilitation interventions spanned all three self-care systems and included repositioning, passive and active-assisted mobilisation, exercise prescription, electrical stimulation, pressure-relief manoeuvres, and education for individuals, caregivers, and professionals. Movement and activity enablement emerged as a prominent cross-cutting theme across systems, linking rehabilitation practice with transitions from dependence toward shared and autonomous self-care. **Conclusions**: Within multidimensional pressure ulcer prevention, this mapping identifies rehabilitation workforce interventions as an under-recognised component, contributing through movement enablement and fostering self-care capacity alongside other established preventive domains. Applying Orem’s theory highlights prevention as a dynamic, person-centred process. Given the small and heterogeneous evidence base and the absence of formal quality appraisal, these findings represent a mapping of potential contributions rather than evidence of effectiveness.

## 1. Introduction

Pressure ulcers, also referred to as pressure injuries, remain a significant and persistent challenge in healthcare systems worldwide [1,2]; this manuscript uses the term pressure ulcer (PU) throughout. Globally, PU cases have increased substantially, rising from approximately 300,000 in 1990 to over 645,000 in 2021. Although the age-standardised prevalence rate has slightly decreased, these injuries remain more frequent among individuals aged 60 years and older. Mortality associated with PU has also increased over time, despite a small reduction in age-standardised death rates. Additionally, the overall burden of disease, measured in disability-adjusted life years (DALYs), has grown, with important differences across regions and age groups [2].

In Europe, PU prevalence remains high, with a median rate of 10.8%. The sacral region is the most affected area. While less severe lesions (Categories I and II) are the most frequent, severe cases (Category IV) still represent a notable proportion of occurrences [3].

The pathogenesis of PU is driven by the synergistic effect of multiple risk factors operating across distinct domains. Biomechanical and functional determinants, particularly reduced activity and mobility, are strong predictors, but they act alongside systemic and metabolic determinants, impaired perfusion and oxygenation, sensory impairment, skin status and microclimate, comorbidity, and advanced age [4,5]. These injuries are associated with substantial physical, psychological, and social consequences for individuals, as well as increased healthcare utilisation, prolonged hospitalisation, and elevated service and organisational costs [6,7].

Among these systemic determinants, nutritional status is a well-established and modifiable contributor to PU risk. A systematic review and meta-analysis reported more than threefold higher odds of PU development in malnourished compared with well-nourished adults (odds ratio 3.66, 95% confidence interval 2.77–4.83). However, most of the pooled studies were appraised as being at moderate risk of bias [8]. Protein-energy deficits and deficiencies in micronutrients such as vitamin C, zinc, and other trace elements may impair tissue integrity, immune competence, and soft-tissue tolerance to mechanical loading [9,10]. Accordingly, international guidance positions nutritional screening and assessment, individualised nutritional planning, adequate hydration, and, where habitual intake is insufficient, protein-energy supplementation as integral components of preventive care [11,12]. Evidence on the comparative effectiveness of specific supplementation strategies nevertheless remains uncertain [13]. PU prevention is therefore multidimensional, and mobility and activity constitute one major modifiable domain within a broader constellation of systemic, metabolic, tissue-related, and environmental determinants. Within this multidimensional preventive landscape, rehabilitation addresses a distinct but interconnected domain, targeting mobility, activity, functional capacity, positioning, and participation in self-care.

Despite advances in prevention and care, PU continue to occur across different care settings, especially among individuals with impaired mobility, reduced sensory perception, or limited capacity for self-care [14,15].

Importantly, certain clinical transitions represent periods of increased risk. For example, individuals with complete spinal cord injuries show a high incidence of PU, particularly during the transition from acute care to inpatient rehabilitation, suggesting that early-stage complications may influence long-term skin integrity outcomes [16].

In this context, effective prevention requires more than isolated clinical actions or devices; rather, it depends on coordinated, sustained, and context-sensitive interventions delivered by healthcare teams [4,17,18].

Rehabilitation is inherently person-centred and function-oriented, aiming to optimise physical capacity, promote independence, and support participation in daily activities [19,20]. The rehabilitation workforce contributes to PU prevention through interventions that address both intrinsic risk factors, such as impaired mobility, reduced activity, and functional decline, and extrinsic factors such as pressure exposure and environmental conditions [4,21].

Although clinical guidelines commonly address PU prevention, much of the literature emphasises risk assessment and repositioning protocols or focuses on specific preventive technologies [4,18]. In contrast, rehabilitation interventions are often described in fragmented or implicit ways, scattered across studies with different aims, populations, and outcomes. This fragmentation makes it challenging to understand how rehabilitation interventions contribute to PU prevention, how they are operationalised in practice, and how responsibilities are distributed between professionals and individuals.

Furthermore, PU prevention is dynamic and closely linked to an individual’s evolving functional status [22,23]. As mobility, activity levels, strength, endurance, and cognitive capacity change over time, so too does the required level of assistance. This variability highlights the importance of conceptual frameworks that can capture gradations of dependence, participation, and self-care. Without such frameworks, preventive interventions risk being conceptualised as static or uniform, rather than responsive to individual capacity and context.

Orem’s Self-Care Deficit Theory provides a theoretically robust and clinically relevant framework for understanding care delivery in relation to self-care capacity [24]. Central to the theory is the assumption that individuals have the potential for self-care but may experience limitations that require nursing or other professional support. Orem conceptualised care delivery through three nursing systems: the wholly compensatory system, in which care is provided entirely by professionals; the partly compensatory system, in which care is shared between professionals and individuals; and the supportive–educative system, in which professionals focus on teaching, guiding, and supporting individuals to perform self-care independently [24]. Applying Orem’s theory to PU prevention allows for a nuanced understanding of how interventions shift across these systems as individuals recover function, adapt to disability, or transition between care settings.

Existing reviews have mapped nutritional support and care integration across settings [11,13,17], while clinical guidelines address risk assessment, repositioning protocols, and support surfaces [4,12,18]. To date, however, no review has explicitly foregrounded rehabilitation as a distinct domain of practice within PU prevention, nor has it systematically examined how rehabilitation-focused interventions align with individuals’ self-care capacity. Consequently, the role of the rehabilitation workforce may remain underrepresented or conceptually diffuse within the broader prevention discourse. Related evidence from chronic wound care shows that formal consensus methods have been used to define interventions relevant to rehabilitation in the treatment of venous and diabetic foot ulcers, including therapeutic exercise, mobility-related approaches, compression therapy, and assistive or offloading strategies [25,26]. Although these studies concern the treatment of established wounds rather than PU prevention, they illustrate the broader relevance of rehabilitation-oriented approaches within multidisciplinary wound care.

Against this background, this scoping review aims to systematically map rehabilitation interventions described in the literature for PU prevention across healthcare settings. Consistent with this aim, the review deliberately addresses the rehabilitation component of prevention rather than comprehensively mapping all preventive interventions; other preventive domains fall outside its scope, although they remain essential elements of multidisciplinary prevention.

## 2. Methods

This scoping review was conducted in accordance with the methodological framework proposed by Arksey and O’Malley [27], further refined by Levac et al. [28], which structured the conduct of the review across five stages: identifying the research question; identifying relevant studies; study selection; charting the data; and collating, summarising, and reporting the results. Reporting followed the Preferred Reporting Items for Systematic Reviews and Meta-Analyses extension for Scoping Reviews (PRISMA-ScR) checklist [29], completed and provided as Supplementary File S1; the Framework Method described by Gale et al. [30] structured the synthesis of the charted data; and Orem’s Self-Care Deficit Theory [24] served as the theoretical lens for categorising and interpreting the identified interventions. No review protocol was registered for this scoping review.

### 2.1. Identifying the Research Question

The following research question guided the review and was developed using the PCC (Population, Concept, Context) mnemonic to ensure alignment with the study objectives and the eligibility criteria:

What rehabilitation interventions are described in the literature for the prevention of PU in people receiving healthcare?

### 2.2. Identifying Relevant Studies

The literature search was conducted across the following electronic databases: Embase, PubMed, Scopus, MEDLINE Complete, Cochrane Central Register of Controlled Trials, Cochrane Database of Systematic Reviews, CINAHL Complete, and Nursing & Allied Health Collection: Comprehensive. These databases were selected to provide broad coverage of the literature on PU, rehabilitation, and medical and nursing interventions. The searches were conducted between 11 and 13 December 2025. No dedicated grey literature search was undertaken; the search was restricted to the eight electronic databases listed above, and no additional searches of grey literature repositories, organisational websites, theses or dissertations, or conference proceedings were conducted. The final search was completed on 13 December 2025, and the evidence base therefore reflects records retrievable in the searched databases up to that date. The upper publication-year limit of 2026 refers to the predefined eligibility window applied to the search and should not be interpreted as indicating temporal coverage beyond the final search date or complete coverage of literature published during 2026.

The search strategy was structured according to the Population, Concept, and Context components of the review question.

Table 1 presents the search terms used to develop the search strings. Terms were derived from Medical Subject Headings (MeSH) and were applied as controlled vocabulary in MEDLINE and PubMed, and as free-text keywords in the remaining databases.

Within each PCC component, terms were combined using the Boolean operator OR, and the Population, Context, and Concept components were subsequently combined using AND. The same term set and Boolean structure were applied independently in each of the eight electronic databases searched. Supplementary File S3 provides the full Boolean search structure, the limits applied, and the number of records retrieved from each database.

### 2.3. Study Selection

Rehabilitation was understood, in accordance with the World Health Organization, as a set of interventions designed to optimise functioning and reduce disability in individuals with health conditions in interaction with their environment [20]. For this review, an intervention was considered part of rehabilitation when the source situated it within rehabilitation practice, a rehabilitation programme or service, or rehabilitation-oriented care. This operationalisation allowed the review to include both interventions directed primarily towards functioning and mobility and preventive components embedded within rehabilitation care. Activities such as repositioning, education, massage, or support-surface use were therefore not classified as rehabilitation interventions solely based on their content but according to how they were situated and described within the source.

The term “rehabilitation workforce” is used as an umbrella term for the healthcare professionals and teams involved in rehabilitation as characterised by the included sources. It does not imply that the mapped interventions are exclusive to particular professional groups, since professional roles and scopes of practice vary across healthcare systems.

Table 2 summarises the eligibility criteria applied in this review.

The criteria also restricted inclusion to full-text articles published between 2015 and 2026 in English or Portuguese. The date limiter was applied to ensure the inclusion of studies reflecting current developments in PU prevention and rehabilitation strategies. Given the ongoing evolution of concepts, guidelines, and interventions related to PU prevention, we aimed to capture the most relevant and up-to-date evidence aligned with contemporary clinical practice.

All retrieved citations were uploaded to Rayyan, (Rayyan Systems, Inc., Cambridge, MA, USA), an AI-assisted platform for systematic review management, where duplicates were detected and removed. Two reviewers screened the titles and abstracts of the remaining records independently against the eligibility criteria. The full texts of potentially eligible records were then retrieved and assessed independently by the same two reviewers. At both stages, the two reviewers discussed disagreements and, where they did not reach consensus, referred them to a third reviewer external to the research team, whose decision was final. Consistent with the purpose of a scoping review, no critical appraisal of the methodological quality of the included sources was undertaken, since the objective was to map the range and nature of the available evidence rather than to appraise the strength of individual findings [29].

**Figure 1:**
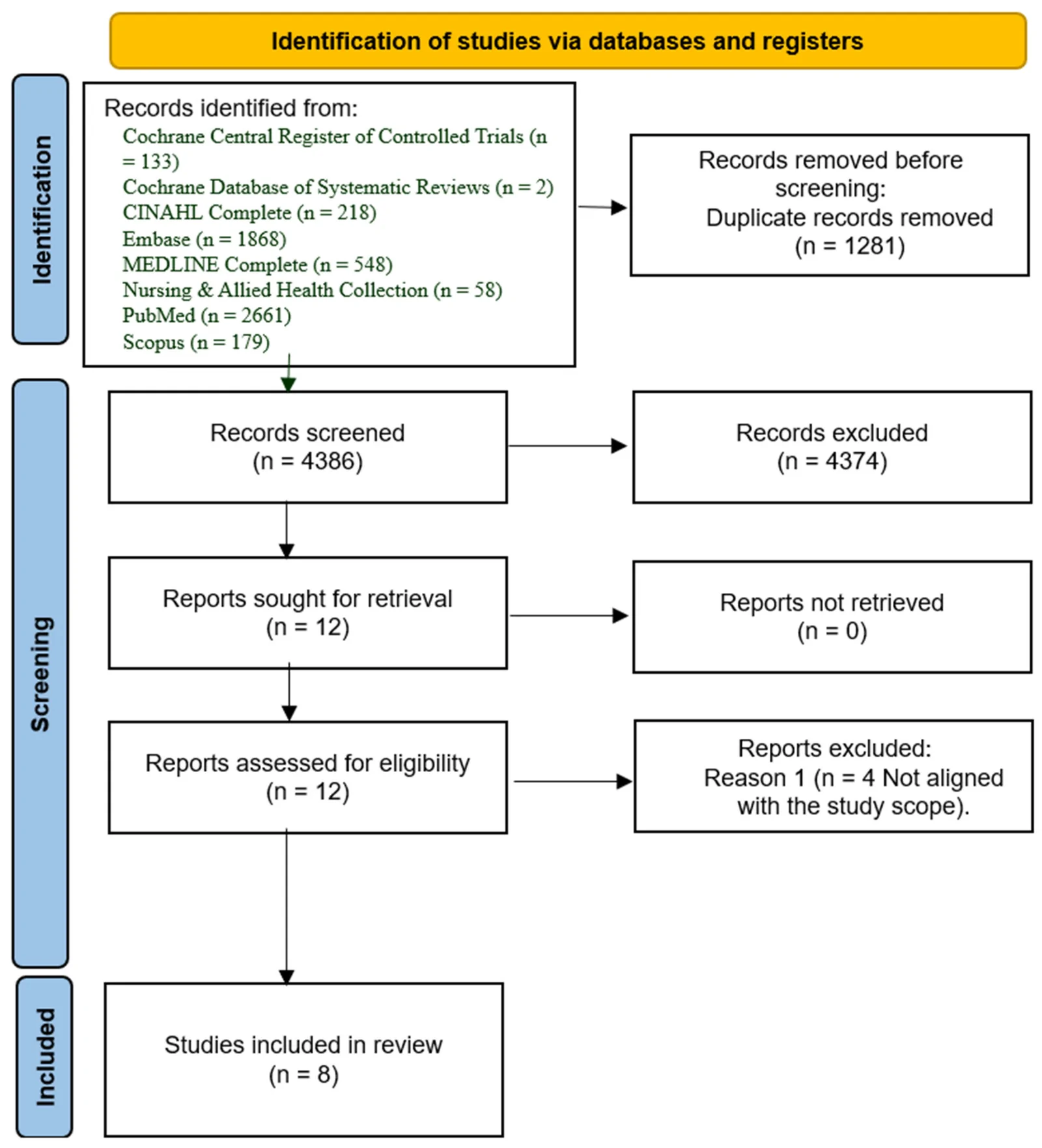
PRISMA flowchart.

The selection process, from the initial search to the final inclusion of studies, is summarised in the PRISMA-ScR flow diagram (Figure 1).

### 2.4. Charting the Data

Data from eligible studies were charted using a purpose-built, structured form developed for this scoping review. The form was designed to capture core bibliographic and study characteristics, including:

- Author(s) and year of publication;
- Country of origin;
- Study design and stated aim;
- Intervention characteristics and key operational components;
- Features mapping to one or more systems within Orem’s self-care deficit theory [24].

Two reviewers conducted the charting and independently extracted and coded information based on a qualitative interpretation of the intervention descriptions reported in each study. Mapping fields informed by Orem’s systems were embedded in the charting form to support subsequent classification of each operational component within the wholly compensatory, partly compensatory, and supportive–educative systems. Reviewers resolved discrepancies through discussion. Both primary and secondary sources were eligible because the review aimed to map rehabilitation interventions described in the literature rather than estimate intervention effectiveness. Source type was recorded during charting and remained identifiable throughout the synthesis, so that primary and secondary sources contributed to the same intervention mapping while their status remained transparent. No evidential weighting was applied to either source type, since the review draws no inference about the strength of the evidence supporting any intervention. To confirm that the included evidence base was not represented twice, the reference list of the secondary source was checked against the primary records retained in the review.

### 2.5. Collating, Summarising, and Reporting the Results

Orem’s Self-Care Deficit Theory was adopted as the framework for categorising the charted interventions. It was not used to formulate the research question, the search strategy, or the eligibility criteria, which were structured using the PCC mnemonic; it was applied after study selection, as a theory-informed framework for charting, categorising, and interpreting the interventions identified. The theory distinguishes three nursing systems—wholly compensatory, partly compensatory, and supportive–educative—according to the extent to which individuals can meet their own self-care requisites [24]. This distinction was considered appropriate for the present synthesis because preventive requirements shift as functional status evolves, and because the theory makes explicit how responsibility for preventive action is distributed between professionals, individuals, and caregivers. Frameworks that describe functioning and disability comprehensively do not model this distribution of responsibility, which is central to interpreting rehabilitation interventions across care settings and levels of dependence.

For the purposes of this synthesis, system assignment was operationalised according to the mode of delivery of each intervention component and the level of active participation required from the individual in that specific component. Self-care capacity provided the theoretical basis for interpreting this distribution of preventive responsibility but was not treated as a separate measurable criterion where it was not explicitly reported by the source. Components performed for the individual without requiring their active participation were coded as wholly compensatory. Components in which the individual actively performed part of the preventive action with professional assistance, supervision, or reinforcement were coded as partly compensatory. Components centred on teaching, guidance, or training to enable subsequent performance of the preventive action were coded as supportive–educative; for this theory-informed synthesis, this category also encompassed educational interventions directed to caregivers or other care providers responsible for implementing preventive care. Where the same intervention type was described with different modes of delivery or levels of participation, across or within sources, it was coded once at each level, which explains why certain intervention types appear in more than one system. A mapping matrix linking each source to the extracted intervention, its operational description, and the assigned system is provided in Supplementary File S2.

To collate, summarise, and report the results, we followed the Framework Method described by Gale et al. [30], using Orem’s Self-Care Deficit Theory [24] as an interpretive lens to guide mapping and synthesis. After familiarisation with the extracted data, two reviewers initially coded intervention descriptions and operational components. A working analytical framework was then developed iteratively, combining inductive codes with theory-informed categories aligned to Orem’s systems (wholly compensatory, partly compensatory, and supportive–educative). The framework was systematically applied to all included records, and the data were charted in a matrix to enable cross-study comparison. Finally, patterns were identified and synthesised by intervention type and by the Orem system, and the findings were reported narratively, supported by summary tables aligning the interventions across studies and by the mapping matrix provided in Supplementary File S2.

## 3. Results

In total, 5667 records were retrieved from the databases. After removing duplicates, 4386 titles and abstracts were screened. Twelve full-text articles were assessed for eligibility, and eight records were ultimately included (Table 3). Seven of these are primary sources, one of which is a trial protocol, and one is a secondary review [23]. None of the seven primary records was cited in the secondary source; no direct overlap between the included primary records and the secondary review was therefore identified. Source type is identified for every charted component in Supplementary File S2.

Consistent with the review objective, the findings are presented as a mapping of the rehabilitation interventions identified, their operational components, and their distribution across the three self-care systems of Orem’s framework.

### 3.1. Wholly Compensatory System

Within the wholly compensatory nursing system, the rehabilitation workforce implemented interventions in which the preventive action was performed for the individual, without requiring their active performance in that specific component.

Patient positioning optimisation, in bed or seated, and scheduled repositioning were described as wholly compensatory strategies to redistribute pressure, reduce prolonged ischaemia over bony prominences, and prevent secondary complications related to immobility and inactivity.

Repositioning was described through structured schedules and included optimisation of alignment in bed and seated positions [21,23,31,32,33].

Another mapped intervention was passive mobilisation to preserve joint range of motion, mitigate stiffness, and prevent contractures. These interventions included passive movements of upper and lower limb joints (e.g., shoulder, elbow, hip, knee, and ankle). They were used to maintain mobility in individuals unable to participate actively in movement [32,33].

As part of wholly compensatory care, limb massage was described as being provided at scheduled intervals to mitigate muscle atrophy and support local circulation in critically ill individuals [33].

The prescription, selection, and management of pressure-redistributing support surfaces were also described as wholly compensatory interventions, including high-specification mattresses, alternating pressure air mattresses, wheelchair cushions, and other therapeutic support surfaces. These interventions were described as part of best-practice protocols to reduce tissue stress and PU risk. Surface selection was individualised based on clinical factors (e.g., body weight, mobility, moisture risk) and required periodic reassessment. Proper fitting of seating systems was emphasised to minimise shear forces, abnormal pressure points, and postural instability [21,23].

Finally, electrical stimulation was described as a wholly compensatory intervention intended to promote pressure redistribution and improve tissue oxygenation. Stimulation targeted large muscle groups, particularly the gluteal muscles, and was delivered without requiring active performance from the individual, using surface electrodes placed directly on the skin or integrated into a form-fitted garment. Stimulation parameters, including frequency, pulse duration, and activation–rest cycles, were predefined and individualised by trained professionals to ensure effective muscle contractions while maintaining safety and comfort. Electrical stimulation was administered for extended daily periods with continuous skin monitoring [34].

### 3.2. Partly Compensatory System

Within the partly compensatory nursing system, the rehabilitation workforce implemented interventions in which individuals actively participated in their care, with professional support, guidance, and supervision to enhance functional capacity and prevent PU.

Exercise-based interventions were prescribed and supervised to increase muscle strength, improve joint range of motion, and enhance trunk stability—key prerequisites for transfers and other functional mobility tasks. Programmes included upper- and lower-limb strengthening (e.g., arm flexion/extension/lifting and knee flexion/extension), with training intensity and duration progressively adjusted according to tolerance and clinical stability. By improving mobility and reducing immobility-related complications, these interventions were described as contributing to PU prevention [21,31,32,33], with one source describing their role in mitigating PU risk [21].

To facilitate early functional recovery, individuals were assisted with bed mobility activities, including bridging, rolling, sitting up with assistance, and active-assisted mobilisations [33] and balance training [21]. When haemodynamically stable, they were supported in adopting upright positions and performing additional lower limb activities, such as leg-swinging exercises, to promote mobility and mitigate risks associated with prolonged immobility [33].

In wheelchair users, assisted pressure-relief manoeuvres were described, with individuals performing scheduled pressure-relieving actions—such as forward leans, lateral shifts, or wheelchair push-ups—every 15 to 30 min. Although these manoeuvres are intended to be self-initiated, supervision and reinforcement were emphasised, given users’ tendency to overestimate pressure-relief frequency, highlighting the need for monitoring and ongoing education [23].

In community-based, training-oriented rehabilitation programmes, mobility-focused exercises requiring active participation were described, delivered under professional supervision to ensure safety and appropriate execution. These interventions were designed to enhance functional movement and prevent secondary complications, including PU, in individuals with partial self-care capacity [21,35].

**Table 3:** Characteristics of the included sources (*n* = 8): authors, year, country, design, aim, and rehabilitation interventions reported.

Authors/Year/Country	Design/Aim	Interventions
Ahmetović et al. [34], 2015 Canada	Feasibility study To investigate the safety, feasibility, and acceptability of a novel treatment, intermittent electrical stimulation, for preventing deep tissue injury in different healthcare settings.	- Electrical stimulation
Cunha et al. [21], 2025 Portugal	Observational, descriptive, exploratory, retrospective study To investigate improvements in activity and mobility associated with rehabilitation nursing interventions in people at risk of PU.	- Teach pressure relief manoeuvres - Patient repositioning and seating - Gait training - Exercise prescription, delivery, and supervision - Prescription of support products
Gour-Provencal et al. [32], 2021 Canada	Prospective cohort study To identify factors associated with the occurrence of PU during acute care and with longer length of stay, focusing on modifiable factors that can be addressed and optimised by the acute rehabilitation team.	- Passive mobilisations - Patient repositioning and seating - Exercise prescription, delivery, and supervision
Wijker et al. [36], 2022 The Netherlands	Study protocol for a randomised controlled trial To investigate whether daily electrical stimulation of gluteal and hamstring muscles combined with usual care is more effective in reducing recurrences of PU than usual care alone in individuals with chronic spinal cord injury, who had at least one PU in the previous five years.	- Electrical stimulation
Zhang & Dong [33], 2025 China	Retrospective cohort study To evaluate the effectiveness of integrating the Orem self-care model with early rehabilitation training in mechanically ventilated ICU patients.	- Limb massage - Patient repositioning - Passive and active-assisted mobilisations - Encouragement of active participation in exercises - Education and guidance on self-directed rehabilitation
Peterson et al. [23], 2025 United States of America	Review To examine the development of PU in individuals with impaired mobility, synthesise evidence-based prevention strategies, identify knowledge gaps, and emphasise the need for proactive, interdisciplinary prevention efforts throughout the rehabilitation continuum.	- Patient repositioning - Staff education - Patient and caregiver training - Teach pressure relief manoeuvres - Selection of appropriate pressure-relieving surfaces
Dreier et al. [31], 2025 Switzerland	Qualitative ethnography study To investigate the implementation of interprofessional movement promotion and the understanding, roles, and expectations of the healthcare professionals involved.	- Patient repositioning and seating - Encouraging patients to move - Exercise prescription, delivery, and supervision
Lardizabal-Dofitas et al. [35], 2025 Philippines	Feasibility study To determine the feasibility of using telehealth and distance learning to train healthcare workers and patients in the integrated rehabilitation and prevention of impairments and disabilities from leprosy, lymphatic filariasis, mycetoma, diabetes, PU, and other chronic wounds.	- Training health care workers and persons with disability - Training in oedema massage and compression bandaging - Exercise prescription, delivery, and supervision

### 3.3. Supportive–Educative System

Within the supportive–educative nursing system, the rehabilitation workforce focused on enabling individuals and caregivers to develop the knowledge, skills, and confidence required for self-management, while supporting other care providers to implement preventive actions. In this system, education, guidance, instruction, and verbal reinforcement were directed towards developing self-management skills and supporting increasingly autonomous performance [21,23,31,33,35].

Professionals encouraged individuals to actively participate in bed mobility and functional activities while providing education and guidance on self-directed rehabilitation. This included instruction on upper and lower-limb strengthening exercises, such as arm flexion and extension, lifting, and knee flexion and extension, to foster autonomy and continuity of movement practices beyond supervised sessions [33].

A trial protocol described electrical stimulation as a self-administered preventive intervention for individuals with chronic spinal cord injury living at home. Stimulation parameters were determined once by a professional at baseline, after which the participant applied the stimulation daily at home using a portable stimulator with surface electrodes or a garment with integrated electrodes, and was instructed to adjust the intensity when muscle activation was insufficient [36].

Purposeful movement was further promoted through clear, precise instructions and ongoing verbal support, enabling individuals to understand how and when to move safely within their functional capacity as part of PU prevention strategies [31]. In this context, encouraging movement was framed as an educational process that supported individuals in internalising movement-promoting behaviours. The scope of rehabilitation practice includes instruction and training in joint and muscle exercises, balance and gait training, and transfer and positioning techniques, together with the clinical prescription of walking aids and environmental adaptations [21].

Beyond direct work with individuals, educational interventions extended to caregivers and other professionals. These included training in proper repositioning techniques, the appropriate use of pressure-relieving equipment, the recognition of early signs of skin breakdown, and moisture management. In rehabilitation settings, education was described as interdisciplinary and sustained, particularly in preparation for long-term home care or transitions to assisted living. Engaging individuals and caregivers in shared decision-making and skill-building—such as teaching pressure-relief manoeuvres for wheelchair users and daily skin inspection—was emphasised as a way to empower self-management and promote long-term skin integrity [23].

The TeleRPOID Project further exemplified the supportive–educative system through telehealth-based education and training, including training in oedema massage and compression bandaging for healthcare workers and persons with disability. A structured programme targeted patients, informal caregivers, and healthcare workers using a combination of asynchronous and synchronous sessions. Educational strategies included printed handouts, instructional videos, and live online sessions. In addition to general content on PU and prevention, rehabilitation-specific training, in collaboration with rehabilitation specialists, addressed scar care, manual oedema massage, compression bandaging, and mobility exercises to support sustainable skill development across care settings [35].

**Figure 2:**
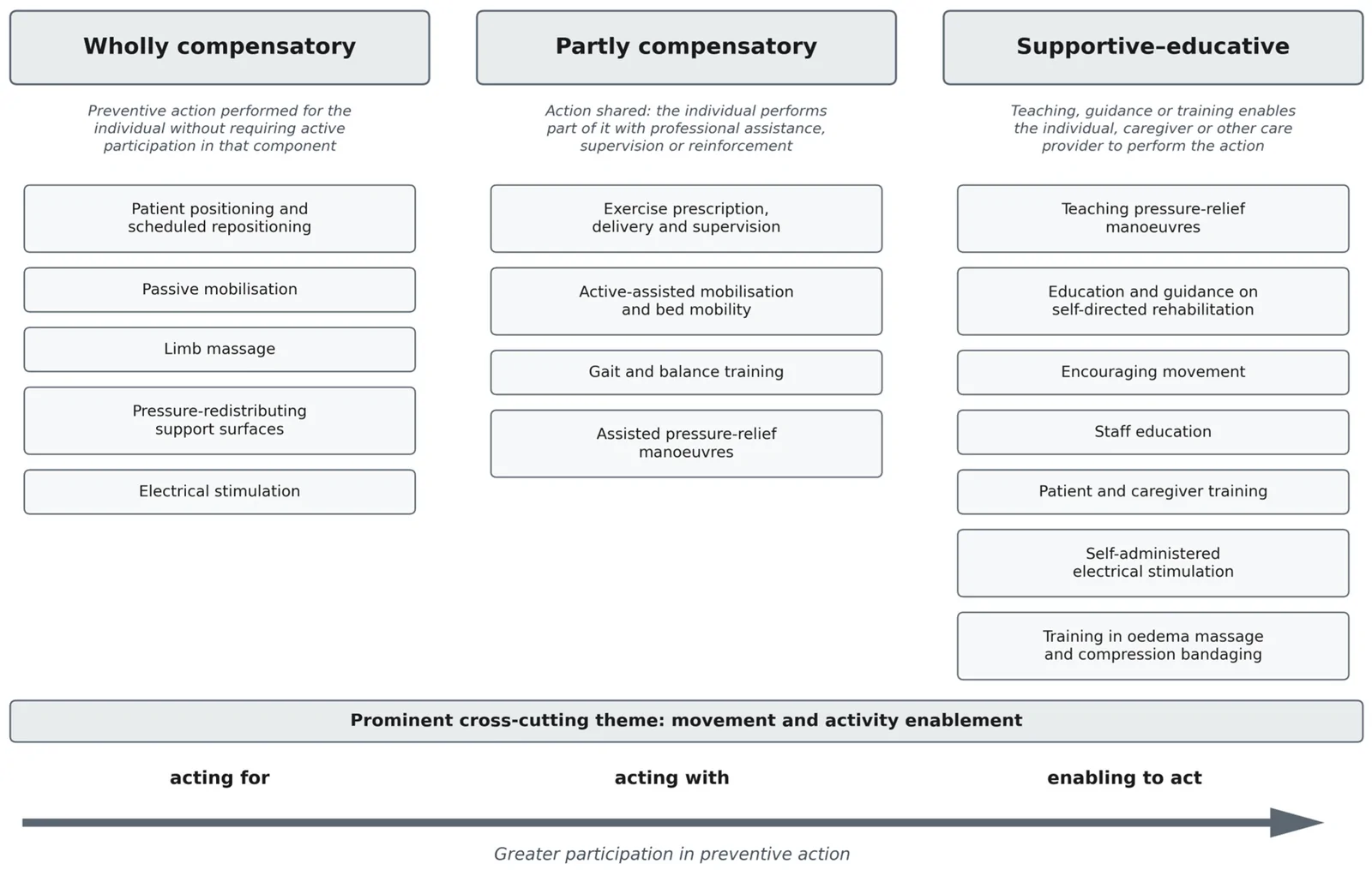
Rehabilitation interventions mapped across Orem’s three self-care systems.

Figure 2 summarises the mapped interventions across the three self-care systems. Movement and activity enablement recurred across all three, in forms that varied with the mode of delivery and level of participation described in the source.

## 4. Discussion

This scoping review offers a theoretically grounded synthesis of rehabilitation workforce interventions for PU prevention, highlighting not only the diversity of strategies employed but also the often-under-recognised contribution of rehabilitation professionals to preventive care. By applying Orem’s self-care deficit theory [24] as an interpretive lens, the review moves beyond a descriptive inventory of interventions. It provides a critical understanding of how prevention is enacted across its three different levels of self-care capacity.

A central finding of this review is that many rehabilitation workforce interventions relevant to PU prevention are not consistently labelled or recognised as PU prevention strategies in the literature. Interventions such as exercise prescription, assisted mobility, movement facilitation, and functional training are frequently framed in terms of rehabilitation outcomes (e.g., strength, transfers, independence). At the same time, their preventive impact on PU development remains implicit.

This lack of explicit recognition contributes to the invisibility of rehabilitation work within prevention discourse, where attention is often directed toward nursing-led repositioning protocols [21,23,31,32,33], or technological solutions [34,35,36]. However, the findings of this review suggest that rehabilitation professionals play a critical role in addressing key biomechanical and functional contributors to PU risk, namely immobility, inactivity, prolonged static loading, and reduced participation in movement, rather than solely managing their consequences. The study by Cunha et al. [21] reports improvements in functional status and mobility/activity levels following rehabilitation nursing interventions.

Importantly, the high prevalence of early-stage PU reported in the literature may signal the need to implement early, proactive protective therapy plans. These findings provide a rationale for further investigation of timely rehabilitation interventions aimed not only at preventing tissue deterioration but also at enhancing functional capacity. This perspective supports a shift from predominantly passive prevention strategies toward a more active, function-oriented approach, where movement and participation are central to risk reduction.

Applying Orem’s framework [24] makes this contribution visible by showing that rehabilitation interventions are not ancillary to prevention but central to supporting transitions from total dependence toward shared and autonomous self-care. This perspective challenges prevention models that prioritise passive strategies and reinforces the need to recognise rehabilitation as a core component of PU prevention pathways.

Importantly, rehabilitation interventions also create opportunities for ongoing visual and tactile skin assessment in high-risk areas and for ensuring pressure relief in vulnerable regions, including the monitoring of early signs such as blanchable erythema [12].

Taken together, the three self-care systems indicate that the distinctive contribution of rehabilitation lies not in any single preventive intervention but in its capacity to adapt preventive action to changing levels of functional independence. Prevention is thereby reframed from a fixed sequence of techniques applied to a person into a progressive redistribution of preventive responsibility that follows that person’s capacity. Although the mapped sources originate from geographically diverse healthcare systems, their heterogeneity and limited number do not support formal comparison between countries or models of care. The common elements identified are better read against contemporary international guidance, which positions PU prevention as multidisciplinary and dependent on the coordinated management of mobility, pressure redistribution, skin integrity, nutrition, and individual capacity [12,18].

Not all individuals will achieve meaningful functional gains, and support surfaces and repositioning schedules remain essential [12]; their contribution may nonetheless be limited where the person remains a passive recipient of care. Rehabilitation-oriented approaches that build strength, balance, endurance, and confidence may improve the ability to reposition, adjust posture, and interrupt prolonged pressure exposure [37,38,39,40], and thereby complement compensatory strategies. Yet movement capacity is seldom framed as a preventive target in the sources mapped in this review. This suggests that prevention frameworks would benefit from widening their focus from pressure management alone to movement enablement, recognising mobility as both a rehabilitative and a preventive goal.

This emphasis on movement enablement should not be interpreted in isolation. Movement addresses the biomechanical and functional dimension of risk. Effective prevention, however, requires its integration with nutritional screening and support, skin and microclimate management, optimisation of perfusion and comorbidity, and appropriate support surfaces [8,11,12]. These domains are interdependent, not merely additive. Muscle mass over bony prominences functions simultaneously as mechanical cushioning and metabolic reserve, so that mobilisation and exercise can sustain or build tissue tolerance only where adequate protein and energy substrate is available. Conversely, rehabilitation programmes themselves increase energy and protein requirements, and prescribing progressive activity without concurrent nutritional assessment may be counterproductive in undernourished individuals [9,10]. Notably, the fragmentation identified in this review is symmetrical. Rehabilitation contributions remain under-recognised within a prevention discourse in which nutrition, although well established in guidance, is seldom integrated with mobility planning. This is so even though nutritional and mobility risk are frequently appraised by the same professional during the same clinical encounter, as in the corresponding subscales of widely used risk assessment instruments. Recognising rehabilitation as a core component of prevention therefore entails situating movement enablement within, rather than apart from, multidimensional and multidisciplinary preventive care, as set out in current international guidance addressing the full range of preventive and therapeutic domains [12].

The supportive–educative system highlights the long-term and often underestimated dimension of PU prevention: enabling individuals and caregivers to sustain preventive behaviours beyond supervised care. Educational interventions targeting movement promotion, pressure-relief techniques, and skin inspection are particularly relevant during transitions from hospital to home or to long-term care, where professional oversight is reduced [4,12,18]. The review, however, reveals that educational and enabling strategies are inconsistently described and often under-theorised. Education is frequently reported as an adjunct to other interventions rather than as a structured, goal-oriented process. From Orem’s perspective, this represents a missed opportunity, as the supportive–educative system is precisely where prevention becomes sustainable and person-centred.

Importantly, enabling self-management should not be understood merely as information transfer. Rehabilitation professionals engage in complex pedagogical work that includes assessing readiness, adapting strategies to cognitive and physical abilities, reinforcing motivation, and supporting behavioural change [41,42]. This work is time-intensive, relational, and context-dependent, which may partly explain why it is less visible in outcome-driven research designs. Nevertheless, this dimension of preventive practice warrants greater recognition and further investigation.

Another critical insight from this review concerns how responsibility for PU prevention is distributed. Although prevention is often framed as a predominantly nursing responsibility [43,44], effective prevention is inherently interprofessional and depends on coordinated input from multiple disciplines. Within this interprofessional approach, rehabilitation professionals contribute distinct expertise in movement analysis, functional progression, and adaptive strategies that complement routine preventive care and may support sustained risk reduction through safer mobility and greater participation.

However, the lack of explicit role delineation in many studies suggests that rehabilitation contributions may be subsumed under broader team activities or remain implicit. This ambiguity risks reinforcing siloed approaches and undervaluing rehabilitation input in prevention planning and evaluation.

### 4.1. Implications for Practice, Research, and Policy

For the person receiving care, the most direct implication is that self-care capacity should determine how prevention is delivered, and that the mode of delivery should change as that capacity changes. Where capacity is absent, prevention is necessarily compensatory; as function returns, the preventive goal should move from acting for the person to acting with them, and then to enabling them to act independently. A person able to learn and perform pressure-relief manoeuvres should not remain a passive recipient of scheduled repositioning indefinitely. Framing prevention in this way makes the transition between levels of assistance an explicit clinical objective rather than an incidental consequence of recovery [12,24].

At the level of the professional team, these findings support involving the rehabilitation workforce in preventive planning from the point of risk assessment, rather than after functional loss or after a PU has developed. Mobility, positioning, pressure-relief techniques, exercise, and the enabling of individuals and caregivers are preventive actions in their own right and can be recorded as such in the care plan. Given the interdependence of preventive domains, this implies clearly defined professional interfaces rather than an expansion of the rehabilitation remit. Rehabilitation professionals are well placed to identify nutritional and skin-related risk during functional assessment and to communicate, refer, and coordinate with other professionals, since the prevention of PU is a team effort [12].

At the organisational and policy level, prevention protocols could incorporate functional status and self-care capacity as determinants of the intensity and nature of preventive action, and documentation systems could capture movement-related preventive activity, which at present remains largely invisible in prevention records. Transitions from hospital to home or to long-term care, where professional oversight is reduced, warrant structured training of the person and the caregiver as part of discharge planning [12,18]. Acknowledging the preventive role of the rehabilitation workforce also has consequences for staffing models, service specification, and training, and the potential economic implications of earlier rehabilitation involvement warrant evaluation in future research. For research, these findings point to designs that examine processes of enablement, transitions between levels of assistance, and the sustainability of preventive behaviours after supervised care ends, rather than intervention efficacy alone. Studies reporting which professional group delivered each preventive component and treating movement or self-care capacity as preventive endpoints rather than solely as rehabilitation outcomes would address two of the principal reporting gaps identified here.

### 4.2. Limitations

The mapping purpose of this review necessarily constrains the conclusions that can be drawn about effectiveness. Because methodological quality and risk of bias were not formally appraised, the presence of an intervention in the mapped literature should not be read as evidence of its comparative effectiveness, nor should the frequency with which an intervention appears be read as a measure of its preventive value. The findings identify patterns of rehabilitation practice and the conceptual relationships between them, rather than establishing the superiority of particular preventive approaches.

The evidence base itself further constrains interpretation. The corpus is small and methodologically heterogeneous, spanning feasibility studies, cohort designs, qualitative research, secondary evidence, and a trial protocol, and the interventions are described at widely differing levels of detail. Although the included sources originate from several healthcare systems, this diversity does not support formal comparison between countries or care models. Given the very small number of sources representing each national context and the marked differences in study design and population, any observed difference could not be attributed to health-system characteristics. Restriction to publications in English or Portuguese, and to the period 2015 to 2026, may have further shaped which healthcare systems are represented, which should be borne in mind when reading the geographical spread of the included sources. No dedicated search of the grey literature was undertaken, so that rehabilitation interventions reported in non-indexed sources, such as institutional or professional reports, theses, or other unpublished materials, may not have been captured, potentially limiting the breadth of the mapped evidence.

Finally, rehabilitation roles were frequently implicit within interventions delivered by interprofessional teams, and the level of detail in intervention descriptions varied considerably between sources. The mapping was therefore bounded by how each source characterised its own interventions. Attribution of preventive contributions to distinct professional groups is also context-dependent, as professional scopes of practice and competency frameworks may differ across countries and healthcare systems; similar preventive actions may therefore be undertaken by different professional groups in different contexts. Together with the limited role delineation in the source reporting, this points to a reporting gap in the rehabilitation and PU literature.

Despite these limitations, the review provides a structured and theoretically grounded overview of rehabilitation interventions for PU prevention. It identifies important conceptual, methodological, and reporting gaps to inform future research and practice.

## 5. Conclusions

Across the eight included records, rehabilitation interventions for PU prevention encompassed positioning and repositioning, passive and active-assisted mobilisation, limb massage, exercise and gait- and balance-related interventions, pressure-relief manoeuvres, pressure-redistributing support surfaces, electrical stimulation, and educational or training strategies for individuals, caregivers, and other care providers. These interventions were distributed across Orem’s wholly compensatory, partly compensatory, and supportive–educative systems, with movement and activity enablement emerging as a prominent cross-cutting theme. The evidence mapped in this review positions rehabilitation workforce interventions as a substantial and under-recognised component of PU prevention, contributing not only through compensatory strategies but also, critically, by enabling movement and fostering self-care capacity. Applying Orem’s Self-Care Deficit Theory reveals prevention as a dynamic process that evolves alongside functional recovery and participation. As a mapping review conducted without formal appraisal of methodological quality, it characterises potential contributions and identifies conceptual, methodological, and reporting gaps rather than establishing the effectiveness of individual interventions. Recognising the often-invisible contribution of the rehabilitation workforce is a necessary step towards more person-centred and sustainable prevention strategies.

## Figures and Tables

**Table 1:** Search terms by PCC component.

Variable		Search Terms
Population	Person at risk of developing a PU	PU; Ulcer, Pressure; Bed Sores; Bed Sore; Sore, Bed; Bedsore; Bedsores; Decubitus Ulcer; Decubitus Ulcers; Ulcer, Decubitus; Pressure Sore; Pressure Sores; Sore, Pressure; Decubitus Sore; Decubitus Sores; Sore, Decubitus; Pressure Injury; Injury, Pressure; Pressure Injuries
Context	Prevention	Prevention and control; Preventive measures; Preventive therapy; Prophylaxis; Control; Prevention
Concept	Rehabilitation interventions	Rehabilitation; Physical therapy; Physical Therapy Modalities; Physiotherapy; Physiotherapies; Physical Therapy Techniques; Physical Therapy Technique; Rehabilitation nursing

**Table 2:** Eligibility criteria.

Variable	Inclusion Criteria	Exclusion Criteria
Population	People of any age at risk of developing PU	Studies focusing exclusively on individuals with established PU where the intervention targets treatment rather than prevention.
Concept	Interventions situated within rehabilitation practice, rehabilitation programmes or services, or rehabilitation-oriented care, irrespective of whether they were delivered by members of a specific rehabilitation profession or within an interprofessional team.	Studies describing interventions not situated within rehabilitation practice, rehabilitation programmes or services, or rehabilitation-oriented care, or conducted in non-healthcare settings.
Context	Interventions focused on preventing PU, including strategies to reduce risk factors.	Studies that do not address prevention or that focus exclusively on the treatment of existing PU.
Type of Studies	Primary and secondary research studies, including randomised controlled trials, quasi-experimental studies, non-randomised intervention studies, observational designs, qualitative research, mixed-methods studies, and secondary research such as systematic reviews, scoping reviews, and narrative or integrative reviews.	Opinion papers, editorials, commentaries, or conference abstracts.

## Data Availability

The data that support the findings of this study are available from the corresponding author upon reasonable request.

## Supplementary Materials

The following supporting information can be downloaded at: https://www.mdpi.com/article/10.3390/nursrep16090339/s1, Supplementary File S1: PRISMA-ScR_checklist; Supplementary File S2: Mapping matrix; Supplementary File S3: Search strategy.

## Public Involvement Statement

No public involvement in any aspect of this research.

## Guidelines and Standards Statement

This manuscript was drafted against the Preferred Reporting Items for Systematic Reviews and Meta-Analyses extension for Scoping Reviews (PRISMA-ScR) checklist. A complete list of reporting guidelines can be accessed via the equator network: https://www.equator-network.org/.

## References

[1] Afzali B.L., Albatineh A.N., Hasanpour D.A., Almutairi M., Ghanei G.R. (2020). The incidence of pressure ulcers and its associations in different wards of the hospital: A systematic review and meta-analysis. Int. J. Prev. Med..

[2] Zhang S., Wei G., Han L., Zhong W., Lu Z., Niu Z. (2025). Global, regional and national burden of decubitus ulcers in 204 countries and territories from 1990 to 2021: A systematic analysis based on the global burden of disease study 2021. Front. Public Health.

[3] Moore Z., Avsar P., Conaty L., Moore D.H., Patton D., O’Connor T. (2019). The prevalence of pressure ulcers in Europe: What does the European data tell us? A systematic review. J. Wound Care.

[4] Haesler E., European Pressure Ulcer Advisory Panel, National Pressure Injury Advisory Panel, Pan Pacific Pressure Injury Alliance (2019). Prevention and Treatment of Pressure Ulcers/Injuries: Clinical Practice Guideline.

[5] Gefen A., Brienza D.M., Cuddigan J., Haesler E., Kottner J. (2022). Our contemporary understanding of the aetiology of pressure ulcers/pressure injuries. Int. Wound J..

[6] Padula W.V., Delarmente B.A. (2019). The national cost of hospital-acquired pressure injuries in the United States. Int. Wound J..

[7] Roussou E., Fasoi G., Stavropoulou A., Kelesi M., Vasilopoulos G., Gerogianni G., Alikari V. (2023). Quality of life of patients with pressure ulcer/injuries: A systematic review. Med. Pharm. Rep..

[8] Chen B., Yang Y., Cai F., Zhu C., Lin S., Huang P., Zhang L. (2023). Nutritional status as a predictor of the incidence of pressure injury in adults: A systematic review and meta-analysis. J. Tissue Viability.

[9] Kassym L., Zhetmekova Z., Kussainova A., Semenova Y., Vetrova A., Nurzhan S., Sarbassova G., Akhmetova A., Orazalina A., Uzbekova S. (2025). Pressure ulcers and nutrients: From established evidence to gaps in knowledge. Curr. Med. Chem..

[10] Manley S., Mitchell A. (2022). The impact of nutrition on pressure ulcer healing. Br. J. Nurs..

[11] Cangelosi G., Sacchini F., Biondini F., Mancin S., Morales Palomares S., Ferrara G., Caggianelli G., Sguanci M., Petrelli F. (2025). Nutritional support in the prevention and treatment of pressure ulcers in healthy aging: A systematic review of nursing interventions in community care. Geriatrics.

[12] National Pressure Injury Advisory Panel, European Pressure Ulcer Advisory Panel, Pan Pacific Pressure Injury Alliance (2025). Prevention and Treatment of Pressure Ulcers/Injuries: Quick Reference Guide.

[13] Langer G., Wan C.S., Fink A., Schwingshackl L., Schoberer D. (2024). Nutritional interventions for preventing and treating pressure ulcers. Cochrane Database Syst. Rev..

[14] Aloweni F., Lim S.H., Gunasegaran N., Ostbye T., Ang S.Y., Siow K.C.E. (2024). Community-acquired pressure injuries: Prevalence, risk factors and effect of care bundles—An integrative review. J. Clin. Nurs..

[15] Sardo P.M.G., Teixeira J.P.F., Machado A.M.S.F., Oliveira B.F., Alves I.M. (2023). A systematic review of prevalence and incidence of pressure ulcers/injuries in hospital emergency services. J. Tissue Viability.

[16] Brienza D., Krishnan S., Karg P., Sowa G., Allegretti A.L. (2018). Predictors of pressure ulcer incidence following traumatic spinal cord injury: A secondary analysis of a prospective longitudinal study. Spinal Cord.

[17] Amêndoa D., Gugg L., Gomes C., Diniz C., Ferreira Ó., Baixinho C.L. (2025). Intervention in healthcare teams to promote adherence to the integration of care for people at risk of pressure injuries between hospitals and communities: A scoping review. Int. Wound J..

[18] Registered Nurses’ Association of Ontario (2024). Pressure Ulcer/Injury Management: Risk Assessment, Prevention and Treatment.

[19] Fernandes J.B., Vareta D., Fernandes S., Almeida A.S., Peças D., Ferreira N., Roldão L. (2022). Rehabilitation workforce challenges to implement person-centered care. Int. J. Environ. Res. Public Health.

[20] World Health Organization (2017). Rehabilitation 2030: A Call for Action.

[21] Cunha R., Amaral P.I.S., Silva M.B.O.R., Rodrigues C.M.S., Oliveira G.A. (2025). Intervention of rehabilitation nurse specialists in the risk of pressure ulcers. Port. J. Rehabil. Nurs..

[22] Muir D., McLarty L., Drinkwater J., Bennett C., Birks Y., Broadway-Parkinson A., Cooksey V., Gleeson P., Holland C., Ledger L. (2024). Pressure injury prevention for people with long-term neurological conditions who self-manage care and live at home. J. Tissue Viability.

[23] Peterson A., Fraix M.P., Agrawal D.K. (2025). Preventing pressure injuries in individuals with impaired mobility: Best practices and future directions. J. Surg. Res..

[24] Orem D.E. Nursing: Concepts of Practice 2001.

[25] Medeiros S., Rodrigues A., Costa R. (2024). Physiotherapy intervention in the treatment of venous ulcers: Results from a Delphi panel. J. Vasc. Dis..

[26] Medeiros S., Rodrigues A., Costa R. (2025). Consensus-based physiotherapy interventions for the treatment of diabetic foot ulcers: A Delphi study. Millenium.

[27] Arksey H., O’Malley L. (2005). Scoping studies: Towards a methodological framework. Int. J. Soc. Res. Methodol..

[28] Levac D., Colquhoun H., O’Brien K.K. (2010). Scoping studies: Advancing the methodology. Implement. Sci..

[29] Tricco A.C., Lillie E., Zarin W., O’Brien K.K., Colquhoun H., Levac D., Moher D., Peters M.D.J., Horsley T., Weeks L. (2018). PRISMA extension for scoping reviews (PRISMA-ScR): Checklist and explanation. Ann. Intern. Med..

[30] Gale N.K., Heath G., Cameron E., Rashid S., Redwood S. (2013). Using the framework method for the analysis of qualitative data in multi-disciplinary health research. BMC Med. Res. Methodol..

[31] Dreier J., Staudacher-Preite S., Bosshart H., Hübsch S., Suter P., Panfil E.M. (2025). Role understanding of the interprofessional team in promoting movement for pressure injury prevention: An ethnographic study. J. Tissue Viability.

[32] Gour-Provencal G., Mac-Thiong J.M., Feldman D.E., Bégin J., Richard-Denis A. (2021). Decreasing pressure injuries and acute care length of stay in patients with acute traumatic spinal cord injury. J. Spinal Cord Med..

[33] Zhang L., Dong M. (2025). Effect of Orem self-care model combined with early rehabilitation training on mechanically ventilated patients in ICU: A retrospective cohort study. Risk Manag. Healthc. Policy.

[34] Ahmetović A., Mushahwar V.K., Sommer R., Schnepf D., Kawasaki L., Warwaruk-Rogers R., Barlott T., Chong S.L., Isaacson G., Kim S. (2015). Safety and feasibility of intermittent electrical stimulation for the prevention of deep tissue injury. Adv. Wound Care.

[35] Lardizabal-Dofitas B., Leochico C.F.D., Ortiz Y.R.H., España A.D.L., Turdanes G.G., Rubite J.M.C. (2025). Feasibility of using telehealth for training health care workers and persons with disability on integrated rehabilitation and prevention of chronic wounds. Acta Med. Philipp..

[36] Wijker B.J., de Groot S., van Dongen J.M., van Nassau F., Adriaansen J.J.E., Achterberg-Warmer W.J., Anema J.R., Riedstra A.T., van Tulder M.W., Janssen T.W.J. (2022). Electrical stimulation to prevent recurring pressure ulcers in individuals with a spinal cord injury compared to usual care: Study protocol. Trials.

[37] Li Z., Guo H., Liu X. (2024). What exercise strategies are best for people with cognitive impairment and dementia? A systematic review and meta-analysis. Arch. Gerontol. Geriatr..

[38] Pelletier C. (2023). Exercise prescription for persons with spinal cord injury: A review of physiological considerations and evidence-based guidelines. Appl. Physiol. Nutr. Metab..

[39] Saraiva J., Rosa G., Fernandes S., Fernandes J.B. (2023). Current trends in balance rehabilitation for stroke survivors: A scoping review of experimental studies. Int. J. Environ. Res. Public Health.

[40] Teodoro J., Fernandes S., Castro C., Fernandes J.B. (2024). Current trends in gait rehabilitation for stroke survivors: A scoping review of randomized controlled trials. J. Clin. Med..

[41] Fernandes J.B., Ferreira N., Domingos J., Ferreira R., Amador C., Pardal N., Castro C., Simões A., Fernandes S., Bernardes C. (2023). Health professionals’ motivational strategies to enhance adherence in rehabilitation of people with lower limb fractures: Scoping review. Int. J. Environ. Res. Public Health.

[42] Fernandes J.B., Fernandes S., Domingos J., Castro C., Romão A., Graúdo S., Rosa G., Franco T., Ferreira A.P., Chambino C. (2024). Motivational strategies used by health care professionals in stroke survivors in rehabilitation: A scoping review. Front. Med..

[43] Li Z., Marshall A.P., Lin F., Ding Y., Chaboyer W. (2022). Registered nurses’ approach to pressure injury prevention: A descriptive qualitative study. J. Adv. Nurs..

[44] Samuriwo R., Dowding D. (2014). Nurses’ pressure ulcer related judgements and decisions in clinical practice: A systematic review. Int. J. Nurs. Stud..

