# Supplementary material for: Rehabilitation Workforce Interventions for Pressure Ulcer Prevention: A Scoping Review
Source: Nurs Rep. 2026 Sep 18;16(9):339. doi: 10.3390/nursrep16090339 (PMC13610841; doi:10.3390/nursrep16090339)
Supplement: Supplementary file 1 [file nursrep-16-00339-s001.zip › nursrep-4558275-supplementary.pdf]

Supplementary_S1_PRISMA-ScR_checklist

Preferred Reporting Items for Systematic reviews and Meta-Analyses extension for Scoping Reviews (PRISMA-ScR) Checklist

SECTION	ITEM	PRISMA-ScR CHECKLIST ITEM	REPORTED ON PAGE #
**TITLE**			
Title	1	Identify the report as a scoping review.	1
**ABSTRACT**			
Structured summary	2	Provide a structured summary that includes (as applicable): background, objectives, eligibility criteria, sources of evidence, charting methods, results, and conclusions that relate to the review questions and objectives.	1
**INTRODUCTION**			
Rationale	3	Describe the rationale for the review in the context of what is already known. Explain why the review questions/objectives lend themselves to a scoping review approach.	1-3
Objectives	4	Provide an explicit statement of the questions and objectives being addressed with reference to their key elements (e.g., population or participants, concepts, and context) or other relevant key elements used to conceptualize the review questions and/or objectives.	3
**METHODS**			
Protocol and registration	5	Indicate whether a review protocol exists; state if and where it can be accessed (e.g., a Web address); and if available, provide registration information, including the registration number.	4
Eligibility criteria	6	Specify characteristics of the sources of evidence used as eligibility criteria (e.g., years considered, language, and publication status), and provide a rationale.	5-6
Information sources*	7	Describe all information sources in the search (e.g., databases with dates of coverage and contact with authors to identify additional sources), as well as the date the most recent search was executed.	4
Search	8	Present the full electronic search strategy for at least 1 database, including any limits used, such that it could be repeated.	4-5; S3
Selection of sources of evidence†	9	State the process for selecting sources of evidence (i.e., screening and eligibility) included in the scoping review.	5-6
Data charting process‡	10	Describe the methods of charting data from the included sources of evidence (e.g., calibrated forms or forms that have been tested by the team before their use, and whether data charting was done independently or in duplicate) and any processes for obtaining and confirming data from investigators.	7-8
Data items	11	List and define all variables for which data were sought and any assumptions and simplifications made.	8
Critical appraisal of individual sources of evidence§	12	If done, provide a rationale for conducting a critical appraisal of included sources of evidence; describe	6

SECTION	ITEM	PRISMA-ScR CHECKLIST ITEM	REPORTED ON PAGE #
		the methods used and how this information was used in any data synthesis (if appropriate).	
Synthesis of results	13	Describe the methods of handling and summarizing the data that were charted.	8-10; S2
**RESULTS**			
Selection of sources of evidence	14	Give numbers of sources of evidence screened, assessed for eligibility, and included in the review, with reasons for exclusions at each stage, ideally using a flow diagram.	7-8
Characteristics of sources of evidence	15	For each source of evidence, present characteristics for which data were charted and provide the citations.	8-13
Critical appraisal within sources of evidence	16	If done, present data on critical appraisal of included sources of evidence (see item 12).	N/A
Results of individual sources of evidence	17	For each included source of evidence, present the relevant data that were charted that relate to the review questions and objectives.	8-10
Synthesis of results	18	Summarize and/or present the charting results as they relate to the review questions and objectives.	11-13
**DISCUSSION**			
Summary of evidence	19	Summarize the main results (including an overview of concepts, themes, and types of evidence available), link to the review questions and objectives, and consider the relevance to key groups.	14-16
Limitations	20	Discuss the limitations of the scoping review process.	16-17
Conclusions	21	Provide a general interpretation of the results with respect to the review questions and objectives, as well as potential implications and/or next steps.	17
**FUNDING**			
Funding	22	Describe sources of funding for the included sources of evidence, as well as sources of funding for the scoping review. Describe the role of the funders of the scoping review.	18

JBI = Joanna Briggs Institute; PRISMA-ScR = Preferred Reporting Items for Systematic reviews and Meta-Analyses extension for Scoping Reviews.
* Where *sources of evidence* (see second footnote) are compiled from, such as bibliographic databases, social media platforms, and Web sites.
† A more inclusive/heterogeneous term used to account for the different types of evidence or data sources (e.g., quantitative and/or qualitative research, expert opinion, and policy documents) that may be eligible in a scoping review as opposed to only studies. This is not to be confused with *information sources* (see first footnote).
‡ The frameworks by Arksey and O'Malley (6) and Levac and colleagues (7) and the JBI guidance (4, 5) refer to the process of data extraction in a scoping review as data charting.
§ The process of systematically examining research evidence to assess its validity, results, and relevance before using it to inform a decision. This term is used for items 12 and 19 instead of "risk of bias" (which is more applicable to systematic reviews of interventions) to include and acknowledge the various sources of evidence that may be used in a scoping review (e.g., quantitative and/or qualitative research, expert opinion, and policy document).

From: Tricco AC, Lillie E, Zarin W, O'Brien KK, Colquhoun H, Levac D, et al. PRISMA Extension for Scoping Reviews (PRISMAScR): Checklist and Explanation. Ann Intern Med. 2018;169:467–473. doi: 10.7326/M18-0850.

Supplementary File S2. Mapping matrix

Source	Extracted intervention	Operational description as reported	System assigned	Source type
Ahmetović et al., 2015	Electrical stimulation	Intermittent electrical stimulation applied to large muscle groups via surface electrodes or form-fitted garments; parameters predefined and individualised by trained professionals; delivered to the person without participation.	Wholly compensatory	Primary
Wijker et al., 2022	Electrical stimulation (self-administered)	Trial protocol; no outcome results reported. Parameters are set once by a professional at baseline; the participant then applies daily stimulation at home using a portable stimulator, and is instructed to adjust the intensity when activation is insufficient.	Supportive–educative	Primary
Cunha et al., 2025	Patient repositioning and seating	Scheduled repositioning and optimisation of alignment in bed and seated positions, performed for the person.	Wholly compensatory	Primary
Cunha et al., 2025	Prescription of support products	Prescription, selection and management of pressure-redistributing support products for the person.	Wholly compensatory	Primary
Cunha et al., 2025	Exercise prescription, delivery and supervision	Limb strengthening and range-of-motion programmes prescribed and supervised, with the person actively performing under professional adjustment of intensity and duration.	Partly compensatory	Primary
Cunha et al., 2025	Gait training	Balance and gait training performed by the person with professional assistance and supervision.	Partly compensatory	Primary
Cunha et al., 2025	Teach pressure relief manoeuvres	Instruction and training in muscular-articular exercises, transfer and positioning techniques, and prescription of assistive devices, oriented to autonomous performance.	Supportive–educative	Primary
Gour-Provencal et al., 2021	Passive mobilisations	Passive movements of upper and lower limb joints to preserve range of motion and prevent	Wholly compensatory	Primary

Source	Extracted intervention	Operational description as reported	System assigned	Source type
		contractures in persons unable to participate actively.		
Gour-Provencal et al., 2021	Patient repositioning and seating	Structured repositioning schedules addressed by the acute rehabilitation team.	Wholly compensatory	Primary
Gour-Provencal et al., 2021	Exercise prescription, delivery and supervision	Prescribed and supervised exercise contributing to mobility and to mitigation of pressure injury risk, performed by the person.	Partly compensatory	Primary
Zhang & Dong, 2025	Limb massage	Limb massage performed at scheduled intervals to mitigate muscle atrophy and support local circulation in critically ill persons.	Wholly compensatory	Primary
Zhang & Dong, 2025	Patient repositioning	Scheduled repositioning delivered to mechanically ventilated ICU patients.	Wholly compensatory	Primary
Zhang & Dong, 2025	Passive mobilisations	Passive movements of limb joints in persons unable to participate in movement.	Wholly compensatory	Primary
Zhang & Dong, 2025	Active-assisted mobilisations and bed mobility	Assisted bridging, rolling, sitting up, leg-swinging and upright positioning when haemodynamically stable; performance shared between professional and person.	Partly compensatory	Primary
Zhang & Dong, 2025	Encourage patients to perform exercises	Encouragement of active participation in bed mobility and functional activities, with the person performing.	Partly compensatory	Primary
Zhang & Dong, 2025	Education and guidance on self-directed rehabilitation	Instruction on upper and lower limb strengthening exercises to foster autonomy and continuity of practice beyond supervised sessions.	Supportive–educative	Primary
Peterson et al., 2025	Selection of pressure-relieving surfaces	Selection and fitting of high-specification mattresses, alternating pressure air mattresses and wheelchair cushions; fitting emphasised to minimise shear and postural instability.	Wholly compensatory	Secondary

Source	Extracted intervention	Operational description as reported	System assigned	Source type
Peterson et al., 2025	Patient repositioning	Repositioning described within best-practice protocols to reduce tissue stress.	Wholly compensatory	Secondary
Peterson et al., 2025	Assisted pressure-relief manoeuvres	Scheduled pressure-relieving actions in wheelchair users (forward leans, lateral shifts, push-ups) every 15–30 minutes, self-initiated but supervised and reinforced.	Partly compensatory	Secondary
Peterson et al., 2025	Staff education	Interdisciplinary and sustained education of professionals on repositioning technique, equipment use and recognition of early skin breakdown.	Supportive–educative	Secondary
Peterson et al., 2025	Patient/caregiver training	Shared decision-making and skill-building with individuals and caregivers, including daily skin inspection and pressure relief manoeuvres.	Supportive–educative	Secondary
Dreier et al., 2025	Patient repositioning and seating	Repositioning operationalised through structured schedules within interprofessional movement promotion.	Wholly compensatory	Primary
Dreier et al., 2025	Exercise prescription, delivery and supervision	Prescribed and supervised exercise performed by the person with professional support.	Partly compensatory	Primary
Dreier et al., 2025	Encouraging patients to move	Purposeful movement promoted through clear instructions and ongoing verbal support, enabling the person to understand how and when to move safely.	Supportive–educative	Primary
Lardizabal-Dofitas et al., 2025	Training in oedema massage and compression bandaging	Teletraining and skills instruction in oedema massage and compression bandaging for health workers and persons with disability.	Supportive–educative	Primary
Lardizabal-Dofitas et al., 2025	Exercise prescription, delivery and supervision	Mobility-focused exercises requiring active participation under professional supervision in community-based programmes.	Partly compensatory	Primary

Source	Extracted intervention	Operational description as reported	System assigned	Source type
Lardizabal-Dofitas et al., 2025	Training health care workers and persons with disability	Telehealth programme combining asynchronous and synchronous sessions, printed handouts and instructional videos for patients, informal caregivers and health workers.	Supportive–educative	Primary

Supplementary File S3. Search strategy

Searches were run between 11 and 13 December 2025. Terms were combined with the Boolean operator OR within each PCC component, and the Population, Context, and Concept components were combined with AND. The same term set and Boolean structure were applied in each of the eight databases listed below. Limits applied: publication years 2015–2026; language English or Portuguese; full text.

Search string

("PU" OR "Ulcer, Pressure" OR "Bed Sores" OR "Bed Sore" OR "Sore, Bed" OR "Bedsore" OR "Bedsores" OR "Decubitus Ulcer" OR "Decubitus Ulcers" OR "Ulcer, Decubitus" OR "Pressure Sore" OR "Pressure Sores" OR "Sore, Pressure" OR "Decubitus Sore" OR "Decubitus Sores" OR "Sore, Decubitus" OR "Pressure Injury" OR "Injury, Pressure" OR "Pressure Injuries")

AND ("Prevention and control" OR "Preventive measures" OR "Preventive therapy" OR "Prophylaxis" OR "Control" OR "Prevention")

AND ("Rehabilitation" OR "Physical therapy" OR "Physical Therapy Modalities" OR "Physiotherapy" OR "Physiotherapies" OR "Physical Therapy Techniques" OR "Physical Therapy Technique" OR "Rehabilitation nursing")

Records retrieved per database

Database / platform	Records
PubMed	2,661
Embase	1,868
MEDLINE Complete	548

Database / platform	Records
CINAHL Complete	218
Scopus	179
Cochrane Central Register of Controlled Trials	133
Nursing & Allied Health Collection: Comprehensive	58
Cochrane Database of Systematic Reviews	2
**Total records retrieved**	**5,667**
Duplicates removed	1,281
Records screened (title and abstract)	4,386
